# Retraction: Thousand and one kinase 1 protects MCAO-induced cerebral ischemic stroke in rats by decreasing apoptosis and pro-inflammatory factors

**DOI:** 10.1042/BSR20190749_RET

**Published:** 2026-08-07

**Authors:** Jiahui Li, Zhijie Liu, Liling Wang, Haiyan Xu, Yulin Wang

**Affiliations:** 1 Department of Neonatal Intensive Care Unit, Shandong Provincial Qianfoshan Hospital, Shandong University, Jinan 250014, Shandong, China; 2 Department of Pediatric Cardiology, Shandong Provincial Hospital Affiliated to Shandong University, Jinan 250014, Shandong, China

**Keywords:** apoptosis, Ischemic stroke, MCAO, pro-inflammatory cytokines, TAOK1

This article is being retracted from *Bioscience Reports* at the request of the Editor-in-Chief and the Editorial Board. This follows an internal investigation, which identified various concerns within the article.

Specifically, these include:

- High similarity between the Figure 3B GAPDH bands (lanes 1-3) and the Figure 2 B-actin bands from Zhang et al. 2020 (DOI: 10.1042/BSR20193031)
- High similarity between the Figure 4E Normal panel of this article and the Figure 6B LoVo Hoechst/Blank panel from Li et al. 2019 (DOI: 10.1111/cpr.12632)
- High similarity between Figure 4E OGD panel and the Figure 3B RKO Hoechst/siRNA panel from Li et al. 2019 (DOI: 10.1111/cpr.12632)
- High similarity between the Figure 4E OGD + NC panel and the Figure 8B LoVo Hoechst/siRNA panel from Li et al. 2019 (DOI: 10.1111/cpr.12632)
- Concerns with the western blots of Figure 5B:
  ◦ Potential splice lines in the Bax and Bcl-2 blots
  ◦ High similarity between lanes 4-5 of the upper and lower ERK bands
  ◦ High similarity between the upper and lower p-ERK bands
  ◦ High similarity between the P38 bands (lanes 2-5) and the Figure 5 MEK1 bands (lanes 2-5) of Chen et al. 2022 (DOI: 10.3390/ph15121551)
  ◦ High similarity between the GAPDH bands (lanes 2-5) and the Figure 3B GAPDH bands (lanes 2-5) of Su et al. 2019 (DOI: 10.1002/jcb.29307)

The authors have been contacted with regard to the retraction and have not responded to the Journal's queries or to the concerns. Given the extent of the issues raised, the Editorial Board stands by the decision to retract the article.

